# Evaluation of the efficacy of chemotherapy for tubular carcinoma of the breast: A Surveillance, Epidemiology, and End Results cohort study

**DOI:** 10.1002/cam4.5763

**Published:** 2023-03-06

**Authors:** Yuting Zhao, Na Chai, Shouyu Li, Lutong Yan, Can Zhou, Jianjun He, Huimin Zhang

**Affiliations:** ^1^ Department of Breast Surgery The First Affiliated Hospital of Xi'an Jiaotong University Xi'an China; ^2^ School of Medicine Xi'an Jiaotong University Xi'an China

**Keywords:** breast cancer‐specific survival, chemotherapy, propensity score matching, SEER, tubular carcinoma

## Abstract

**Background:**

The use of systematic treatment for tubular carcinoma (TC) of the breast remained controversial. This study aimed to explore the efficacy of chemotherapy on TC to develop individualized treatment strategies.

**Methods:**

Using the Surveillance, Epidemiology, and End Results (SEER) database, 6486 eligible cases with TC and 309,304 with invasive ductal carcinoma (IDC) were collected. Breast cancer‐specific survival (BCSS) was assessed through multivariable Cox analyses and Kaplan–Meier analyses. Differences between groups were balanced using propensity score matching (PSM) and inverse probability of treatment weighting (IPTW).

**Results:**

Compared with IDC patients, TC patients had a more favorable long‐term BCSS after PSM (hazard ratio = 0.62, *p* = 0.004) and IPTW (hazard ratio = 0.61, *p* < 0.001). Chemotherapy was an unfavorable predictor of BCSS for TC (hazard ratio = 3.20, *p* < 0.001). After stratifying by hormone receptor (HR) and lymph node (LN) status, chemotherapy was correlated with worse BCSS in the HR+/LN− subgroup (hazard ratio = 6.95, *p* = 0.001) but showed no impact on BCSS in the HR+/LN+ (hazard ratio = 0.75, *p* = 0.780) and HR−/LN− (hazard ratio = 7.87, *p* = 0.150) subgroups.

**Conclusions:**

Tubular carcinoma is a low‐grade malignant tumor with favorable clinicopathological features and excellent long‐term survival. Adjuvant chemotherapy was not recommended for TC regardless of HR and LN status, while the therapy regimens should be carefully individualized.

## Graphical abstract

Based on a large population derived from the Surveillance, Epidemiology, and End Results (SEER) database, this study aimed to clarify the clinicopathological characteristics and long‐term prognosis of tubular carcinoma (TC), evaluate the efficacy of chemotherapy for tubular carcinoma of the breast, and stratify TC patients according to different HR and LN status to provide evidence of individual treatment for TC.

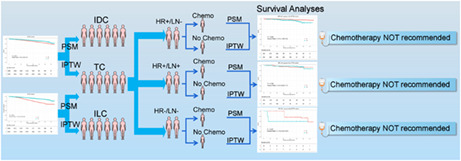

## 1 INTRODUCTION

Breast cancer is a highly heterogeneous disease whose prognosis depends on many factors, including histological subtype, molecular subtype, response to treatment, and so on. The histological subtype is a widely used indicator for therapeutic decision‐making, among which 75% of invasive breast cancer is the subtype of non‐specific invasive ductal carcinoma (IDC), while 25% belong to special histological types. Tubular carcinoma (TC) is one of the nine special subtypes of invasive breast cancer published by the fifth World Health Organization (WHO) Classification of Tumors, accounting for only 1%–4% of invasive breast cancer.^1^, ^2^

Pure breast TC comprises angulated glands with low‐grade nuclei in the desmoplastic stroma. It is pathologically diagnosed by well‐differentiated open tubules compromising over 90% of the tumor, of which the diagnosis relies on complete surgical specimens.^2^, ^3^ Compared with IDC, TC is characterized by smaller tumor size, fewer involved lymph nodes (LNs), higher expression of estrogen and progesterone receptors (PRs), and so on.^4^ TC is a subtype with low potential malignancy and favorable prognosis and has been classified as one of the favorable histologies by the National Comprehensive Cancer Network (NCCN) guidelines. Invasive lobular carcinoma (ILC) is the second most common subtype of breast cancer, which has some similar clinicopathological features to TC, such as up to 90% of ILCs have positive estrogen receptor (ER) and negative human epidermal growth factor receptor‐2 (HER2), with low malignancy and a favorable prognosis.^5^, ^6^ However, most treatment evidence for these subtypes provided by the NCCN guidelines was derived from studies on IDC due to the rarity of these special subtypes. Given that TC, one of the favorable subtypes of breast cancer, is less invasive and has a better prognosis than IDC, IDC‐based evidence may lead to overtreatment of TC.

Treatment recommendations for TC should be based on its specific clinicopathological characteristics and response to therapies. With the insight into breast cancer and progress in molecular‐level technology, molecular classification has been proposed to refine the treatment for individuals.^7^, ^8^ According to the precedents' refinement of breast cancer, almost every special histological subtype corresponds to one molecular subtype.^9^ As one of the special subtypes of breast cancer, TC also has a unique molecular portrait and requires individual treatment according to its clinicopathological features. As reported previously, TC was characterized by high expression of ER and PR,^10^, ^11^, ^12^ which provided a basis for systemic treatment based on endocrine therapy. However, the efficacy of chemotherapy on TC is still not clear. Previous studies demonstrated that due to the small risk of recurrence and metastasis and favorable long‐term prognosis of TC, chemotherapy was not recommended for TC to avoid the risk of overtreatment.^11^, ^13^, ^14^ Nevertheless, TC patients with positive hormone receptors (HR) and positive LNs were recommended to consider chemotherapy according to NCCN guidelines.

Based on a large population derived from the Surveillance, Epidemiology, and End Results (SEER) database, this study aimed to clarify the clinicopathological characteristics and long‐term prognosis of TC, evaluate the efficacy of chemotherapy for TC and stratify TC patients according to different HR and LN status to provide evidence of individual treatment for them.

## 2 MATERIALS AND METHODS

### 2.1 Population and data collection

**FIGURE 1:**
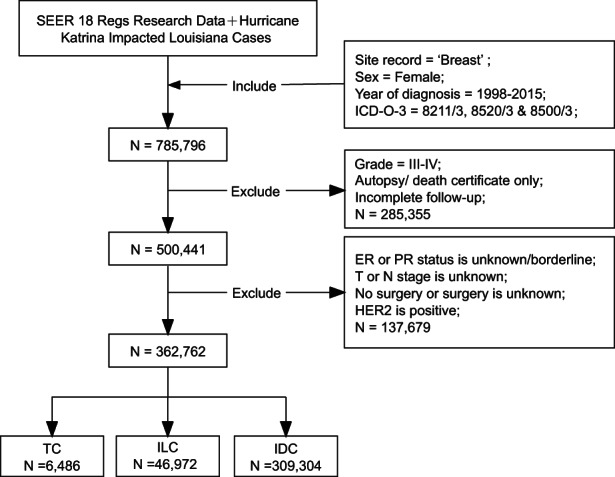
Flowchart of the study design. ER, estrogen receptor; HER2, human epidermal growth factor receptor‐2; ICD‐O‐3, the International Classification of Disease for Oncology, third edition; IDC, invasive ductal carcinoma; ILC, invasive lobular carcinoma; PR, progesterone receptor; SEER, Surveillance, Epidemiology, and End Results database; TC, tubular carcinoma.

Data were accessed from SEER 18 Regs Research with an agreement. Female patients who were diagnosed as TC, IDC, and ILC in 1998–2015 with the primary tumor site code 8211/3: Tubular adenocarcinoma, 8500/3: Infiltrating duct carcinoma, NOS, and 8520/3: Lobular carcinoma in the International Classification of Disease for Oncology, third edition (ICD‐O‐3) were selected from SEER*Stat version 8.3.8. Exclusion criteria: (1) unknown age when diagnosed, histopathologic grade, Breast‐Adjusted American Joint Committee on Cancer (AJCC) sixth T and N stages, length of survival, ER status or PR status; (2) diagnosed with autopsy only or death certificate; (3) incomplete follow‐up; (4) histopathological differentiation grade III or IV; (5) positive HER2 status; (6) no surgical information, or no surgery. Figure 1 shows the design of this study and the selection of subjects. HR status was defined by ER and PR status. The positive ER status or PR status was recognized as positive HR status, while the negative ER status and PR status were recognized as negative HR status.

### 2.2 Endpoint

The endpoint of the current research was breast cancer‐specific survival (BCSS), with the definition of the time from the diagnosis until death from breast cancer.

### 2.3 Statistical analysis

Propensity score matching (PSM) and inverse probability of treatment weighting (IPTW) were used for balancing the differences in clinicopathological characteristics between TC and IDC patients. Logistic regression analysis was used to calculate propensity scores by clinicopathological characteristics, including age at diagnosis, race, marital status, grade, T stage, N stage, HR status, surgery, chemotherapy, and radiotherapy. According to propensity scores, the TC and IDC groups were matched at a ratio of 1:1 using the nearest neighbor matching with a caliper of 0.2. IPTW allocates weights to each study subject according to the calculated propensity scores. The distribution of propensity scores was then made consistent between the two groups, thus controlling for confounding bias. Differences between groups were compared by Pearson's chi‐square test, Fisher's exact test, and standardized mean difference (SMD). An SMD less than 0.1 indicated an excellent balance effect. The same approaches of PSM and IPTW were applied to TC and ILC groups. Then, the TC group was split into three subgroups according to the status of HRs and LNs, including HR‐positive and LN‐negative (HR+/LN−), HR‐positive and LN‐positive (HR+/LN+), and HR‐negative and LN‐negative (HR−/LN−), and each subgroup was divided into a group with chemotherapy (chemotherapy group) and a group with no chemotherapy or chemotherapy information was unknown (no/unknown chemotherapy group). Differences between groups were also balanced using 1:1 PSM and IPTW with the methods mentioned above, respectively. Clinicopathological characteristics for matching included age at diagnosis, race, marital status, grade, T stage, surgery, and radiotherapy.

The Cox hazard model was applied to generate hazard ratios with 95% confidence intervals (CIs) to evaluate risk factors affecting BCSS. Odds ratios (ORs) with 95% CIs were calculated through logistic regression analysis to evaluate predictors of receiving chemotherapy in the TC group. The Cox hazard model and logistic regression analysis were tested by the likelihood ratio test and Wald test. Survival analyses were performed with the Kaplan–Meier method and log‐rank test. Two‐sided *p* < 0.05 was used to show statistical significance. The software package R version 4.1.3 (www.r‐project.org/) was applied to analyze all the statistics.

## 3 RESULTS

### 3.1 Participants

This study involved 362,762 subjects, including 6486 patients diagnosed with TC, 46,972 with ILC and 309,304 with IDC. Table 1 revealed the clinicopathological characteristics of the cohorts of TC and IDC. TC and IDC groups had significantly different characteristics: TC patients were more likely to be diagnosed between 40 and 64 years old (*n* = 3958, 61%), well‐differentiated (grade I) (*n* = 5982, 92.2%), T0–1 stage (*n* = 6223, 95.9%), N0 stage (*n* = 6105, 94.1%), positive HR status (*n* = 6404, 98.7%), treated with mastectomy (*n* = 5024, 77.5%) and radiotherapy (*n* = 3810, 58.7%). Chemotherapy was performed in 7.6% (*n* = 494) of TC patients and 27.9% (*n* = 86,436) of IDC patients. The differences in the clinicopathological features between TC and IDC patients were eliminated after 1:1 PSM and IPTW. As shown in Table S1, ILC also revealed different characteristics compared to TC, that ILC had tumor that was less differentiated (grade II) (*n* = 31,357, 66.8%), larger in size (T2–4 stage) (*n* = 21,584, 46%), more LNs involved (N1–3 stage) (*n* = 15,025, 32%), and ILC patients received more chemotherapy as well (*n* = 14,989, 31.9%). However, due to the small sample size of ILC, the PSM approach failed to balance the features between ILC and TC (*p* < 0.001, SMD > 0.1), thus a better balance was obtained by the further application of IPTW (*p* > 0.05, SMD < 0.1).

**TABLE 1:** Clinicopathological characteristics of breast cancer patients diagnosed with IDC or TC in the original cohort, PSM cohort, and IPTW cohort.

Covariates	Original cohort				PSM cohort				IPTW cohort			
	TC (%)	IDC (%)	*p* value	SMD	TC (%)	IDC (%)	*p* value	SMD	TC (%)	IDC (%)	*p* value	SMD
	*n* = 6486	*n* = 309,304			*n* = 6486	*n* = 6486			*n* = 6486	*n* = 6484		
Age at diagnosis			<0.001	0.184			0.368	0.019			1.000	<0.001
<40	105 (1.6)	10,364 (3.4)			105 (1.6)	91 (1.4)			105 (1.6)	105 (1.6)		
40–64	3958 (61.0)	163,735 (52.9)			3958 (61.0)	4021 (62.0)			3958 (61.0)	3956 (61.0)		
≥65	2423 (37.4)	135,205 (43.7)			2423 (37.4)	2374 (36.6)			2423 (37.4)	2423 (37.4)		
Race			<0.001	0.233			0.576	0.011			0.999	0.001
White	5476 (84.4)	232,358 (75.1)			5476 (84.4)	5451 (84.0)			5476 (84.4)	5474 (84.4)		
Black	310 (4.8)	23,397 (7.6)			310 (4.8)	336 (5.2)			310 (4.8)	310 (4.8)		
Others	700 (10.8)	53,549 (17.3)			700 (10.8)	699 (10.8)			700 (10.8)	700 (10.8)		
Marital status			<0.001	0.074			0.877	0.006			0.999	0.001
Married	3882 (59.9)	173,842 (56.2)			3882 (59.9)	3855 (59.4)			3882 (59.9)	3882 (59.9)		
Unmarried/loss of marriage	2354 (36.3)	122,889 (39.7)			2354 (36.3)	2382 (36.7)			2354 (36.3)	2353 (36.3)		
Unknown	250 (3.9)	12,573 (4.1)			250 (3.9)	249 (3.8)			250 (3.9)	249 (3.8)		
Grade			<0.001	1.536			0.819	0.005			0.998	<0.001
I	5982 (92.2)	103,102 (33.3)			5982 (92.2)	5974 (92.1)			5982 (92.2)	5980 (92.2)		
II	504 (7.8)	206,202 (66.7)			504 (7.8)	512 (7.9)			504 (7.8)	504 (7.8)		
T stage			<0.001	0.594			0.730	0.011			0.998	0.001
0–1	6223 (95.9)	235,978 (76.3)			6223 (95.9)	6238 (96.2)			6223 (95.9)	6221 (95.9)		
2	221 (3.4)	62,365 (20.2)			221 (3.4)	205 (3.2)			221 (3.4)	221 (3.4)		
3–4	42 (0.6)	10,961 (3.5)			42 (0.6)	43 (0.7)			42 (0.6)	42 (0.6)		
N stage			<0.001	0.564			0.972	0.013			1.000	0.001
0	6105 (94.1)	231,018 (74.7)			6105 (94.1)	6111 (94.2)			6105 (94.1)	6102 (94.1)		
1	351 (5.4)	60,922 (19.7)			351 (5.4)	344 (5.3)			351 (5.4)	352 (5.4)		
2	23 (0.4)	12,437 (4.0)			23 (0.4)	25 (0.4)			23 (0.4)	23 (0.4)		
3	7 (0.1)	4927 (1.6)			7 (0.1)	6 (0.1)			7 (0.1)	7 (0.1)		
HR status			<0.001	0.263			0.937	0.001			0.933	0.001
Positive	6404 (98.7)	290,070 (93.8)			6404 (98.7)	6406 (98.8)			6404 (98.7)	6405 (98.8)		
Negative	82 (1.3)	19,234 (6.2)			82 (1.3)	80 (1.2)			82 (1.3)	81 (1.2)		
Surgery			<0.001	0.313			0.950	0.008			0.959	0.001
Mastectomy	5024 (77.5)	195,960 (63.4)			5024 (77.5)	5020 (77.4)			5024 (77.5)	5021 (77.4)		
BCT	1462 (22.5)	113,344 (36.6)			1462 (22.5)	1466 (22.6)			1462 (22.5)	1463 (22.6)		
Radiotherapy			<0.001	0.086			0.721	0.001			0.968	0.001
No/unknown	2676 (41.3)	140,834 (45.5)			2676 (41.3)	2697 (41.6)			2676 (41.3)	2674 (41.2)		
Yes	3810 (58.7)	168,470 (54.5)			3810 (58.7)	3789 (58.4)			3810 (58.7)	3810 (58.8)		
Chemotherapy			<0.001	0.552			0.194	0.005			0.953	0.001
No/unknown	5992 (92.4)	222,868 (72.1)			5992 (92.4)	5951 (91.8)			5992 (92.4)	5989 (92.4)		
Yes	494 (7.6)	86,436 (27.9)			494 (7.6)	535 (8.2)			494 (7.6)	495 (7.6)		

Abbreviations: BCT, breast‐conserving therapy; HR, hormone receptor; IDC, invasive ductal carcinoma; IPTW, inverse probability of treatment weighting; PSM, propensity score matching; SMD, standardized mean difference; TC, tubular carcinoma.

### 3.2 Survival analyses

**FIGURE 2:**
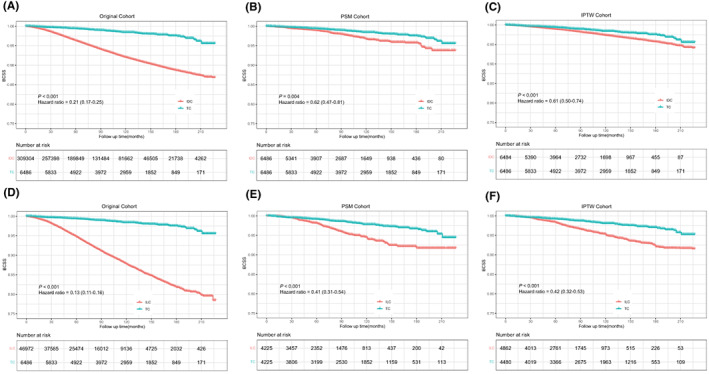
Comparison of BCSS among TC, IDC, and ILC. The Kaplan–Meier methods and log‐rank tests based on the original cohort (A), PSM cohort (B), and IPTW cohort (C) between TC and IDC, and those based on the original cohort (D), PSM cohort (E), and IPTW cohort (F) between TC and ILC. BCSS, breast cancer‐specific survival; IDC, invasive ductal carcinoma; ILC, invasive lobular carcinoma; IPTW, inverse probability of treatment weighting; PSM, propensity score matching; TC, tubular carcinoma.

Median follow‐up was 6.67 years (range, from 0 months to 18.92 years). As shown in Figure 2, TC had a more favorable long‐term BCSS than IDC in the original cohort (hazard ratio = 0.21, 95% CI: 0.17–0.25, *p* < 0.001; Figure 2A), PSM cohort (hazard ratio = 0.62, 95% CI: 0.47–0.81, *p* = 0.004; Figure 2B), and IPTW cohort (hazard ratio = 0.61, 95% CI: 0.50–0.74, *p* < 0.001; Figure 2C). Ten‐year BCSS were 98.4% and 98.4% for TC and 97.3% and 97.4% for IDC in the PSM cohort and IPTW cohort, respectively. Similarly, TC had a better BCSS than ILC in the original cohort (hazard ratio = 0.13, 95% CI: 0.11–0.16, *p* < 0.001; Figure 2D), PSM cohort (hazard ratio = 0.41, 95% CI: 0.31–0.54, *p* < 0.001; Figure 2E), and IPTW cohort (hazard ratio = 0.42, 95% CI: 0.32–0.53, *p* < 0.001; Figure 2F). Ten‐year BCSS was 97.8% and 98.0% for TC and 94.6% and 95.3% for ILC in the PSM cohort and IPTW cohort, respectively. According to the multivariate Cox analysis shown in Table S2, TC subtype was a favorable factor (IDC, hazard ratio = 1.84, 95% CI: 1.51–2.25, *p* < 0.001; ILC, hazard ratio = 2.01, 95% CI: 1.64–2.46, *p* < 0.001) for BCSS in breast cancer after adjusting for other factors. In addition, some unfavorable predictors of BCSS were revealed in the Cox regression analysis, including T2–4 stage (T2, hazard ratio = 2.09, 95% CI: 2.03–2.16, *p* < 0.001; T3–4, hazard ratio = 3.48, 95% CI: 3.32–3.64, *p* < 0.001), LN involvement (N1, hazard ratio = 1.86, 95% CI: 1.79–1.92, *p* < 0.001; N2, hazard ratio = 3.37, 95% CI: 3.21–3.53, *p* < 0.001, N3, hazard ratio = 6.27, 95% CI: 5.96–6.61, *p* < 0.001), and negative HR status (hazard ratio = 2.20, 95% CI: 2.11–2.29, *p* < 0.001).

**FIGURE 3:**
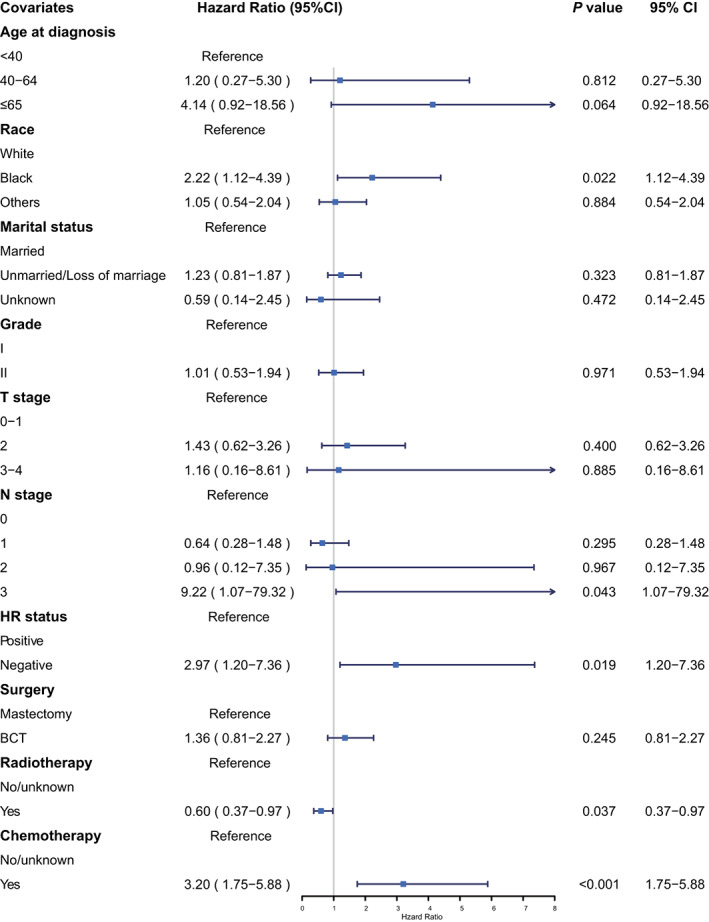
Forest plot for multivariate Cox analysis of predictors in patients with tubular carcinoma. BCSS, breast cancer‐specific survival; BCT, breast‐conserving therapy; CI, confidence interval; HR, hormone receptor.

Then, prognostic risk factors for TC patients were further evaluated. Black race (hazard ratio = 2.22, 95% CI: 1.12–4.39, *p* = 0.022), N3 stage (hazard ratio = 9.22, 95% CI: 1.07–79.32, *p* = 0.043), negative HR status (hazard ratio = 2.97, 95% CI: 1.20–7.36, *p* = 0.019), and chemotherapy (hazard ratio = 3.20, 95% CI: 1.75–5.88, *p* < 0.001) were shown to be independent risk factors for BCSS (Figure 3). The Kaplan–Meier survival analysis was also used to explore the influence of chemotherapy on BCSS in TC patients, which suggested that TC patients with chemotherapy had poorer long‐term survival than those with no/unknown chemotherapy (hazard ratio = 2.38, 95% CI: 1.43–3.97, *p* = 0.001; Figure S1). The 10‐year BCSS was as high as 98.5% in the patients with no/unknown chemotherapy, while 97.4% in those receiving chemotherapy.

### 3.3 Clinicopathological features of patients with TC receiving chemotherapy

The univariate and multivariate analysis demonstrated that chemotherapy was an unfavorable factor of prognosis for TC, so we analyzed the clinicopathological characteristics of TC patients who received chemotherapy. To explore the clinicopathological features of patients who tended to receive chemotherapy, differences were analyzed between TC patients with chemotherapy and those with no/unknown chemotherapy (Table S3). Patients with younger age (age at diagnosis ≤64, *n* = 419, 84.8%), married status (*n* = 325, 65.6%), moderately differentiated tumor (grade II, *n* = 73, 14.88%), advanced T stage (T2–4, *n* = 90, 18.2%), positive LN status (N1–3, *n* = 494, 36.4%), negative HR status (*n* = 14, 2.8%), and breast‐conserving therapy (BCT) (*n* = 171, 34.6%) had a tendency to be treated with chemotherapy. Furthermore, multivariable logistic regression analysis shown in Table 2 revealed that younger age (age at diagnosis 40–64, OR = 0.24, 95% CI: 0.15–0.39, *p* < 0.001; age at diagnosis ≥65, OR = 0.06, 95% CI: 0.04–0.11, *p* < 0.001), black race (OR = 1.30, 95% CI: 1.03–2.35, *p* = 0.03), moderately differentiated (grade II, OR = 1.78, 95% CI: 1.29–2.41, *p* < 0.001), T2 stage (OR = 4.93, 95% CI: 3.44–7.01, *p* < 0.001), positive LNs (N1, OR = 13.48, 95% CI: 10.37–17.53, *p* < 0.001; N2, OR = 24.83, 95% CI: 9.82–66.97, *p* < 0.001; N2, OR = 7.83, 95% CI: 1.40–45.94, *p* = 0.016), and receiving BCT (OR = 1.45, 95% CI: 1.07–1.96, *p* = 0.017) were clinicopathological features of receiving chemotherapy for TC patients.

**TABLE 2:** Clinicopathological features of receiving chemotherapy in patients diagnosed with TC using multiple logistic regression.

Covariates	TC		
	OR	*p* value	95% CI
Age at diagnosis			
<40	Reference		
40–64	0.24	<0.001	0.15–0.39
≥65	0.06	<0.001	0.04–0.11
Race			
White	Reference		
Black	1.58	0.030	1.03–2.35
Others	1.14	0.404	0.83–1.55
Marital status			
Married	Reference		
Unmarried/loss of marriage	0.85	0.161	0.68–1.07
Unknown	0.58	0.107	0.28–1.08
Grade			
I	Reference		
II	1.78	<0.001	1.29–2.41
T stage			
0–1	Reference		
2	4.93	<0.001	3.44–7.01
3–4	1.90	0.180	0.69–4.60
N stage			
0	Reference		
1	13.48	<0.001	10.37–17.53
2	24.83	<0.001	9.82–66.97
3	7.83	0.016	1.40–45.94
HR status			
Positive	Reference		
Negative	3.08	<0.001	1.55–5.70
Surgery			
Mastectomy	Reference		
BCT	1.45	0.017	1.07–1.96
Radiotherapy			
No/unknown	Reference		
Yes	1.10	0.493	0.84–1.47

Abbreviations: BCT, breast‐conserving therapy; CI, confidence interval; HR, hormone receptor; OR, odds ratio; TC, tubular carcinoma.

### 3.4 Prognostic effect of chemotherapy on TC patients with stratification analyses based on HR and LN status

Based on the results that HR and LN statuses were strong prognostic predictors for TC patients, the TC group was then divided into three subgroups of HR+/LN−, HR+/LN+, and HR−/LN− to provide more individualized therapeutic advice, while TC patients with negative HR status and positive LN (HR−/LN+) were not analyzed due to the small sample size (only four patients met the criteria). In each subgroup, significant differences were found between patients with chemotherapy and those with no/unknown chemotherapy. (Table S4). The methods of 1:1 PSM (Table S5) and IPTW (Table S6) were performed within each subgroup to control for confounding bias, which showed good balance results.

**FIGURE 4:**
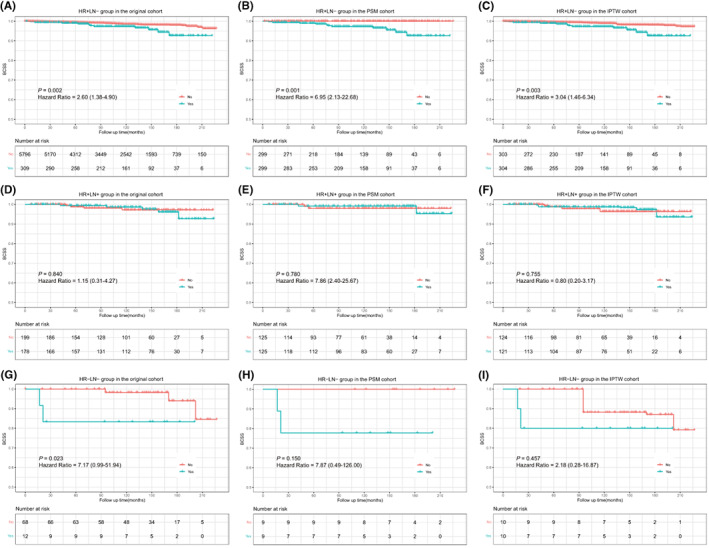
Prognostic effect of chemotherapy on tubular carcinoma patients with different HR and LN status. Prognosis of patients with HR+/LN− (A–C), HR+/LN+ (D–F), and HR−/LN− (G–I) status in the original cohort, PSM cohort, and IPTW cohort, respectively. BCSS, breast cancer‐specific survival; HR, hormone receptor; IPTW, inverse probability of treatment weighting; LN, lymph node; PSM, propensity score matching.

According to Kaplan–Meier survival analyses shown in Figure 4, in the original cohort, TC patients with chemotherapy were associated with a worse BCSS in the HR+/LN− (hazard ratio = 2.60, 95% CI: 1.38–4.90, *p* = 0.002; Figure 4A) and HR−/LN− (hazard ratio = 7.17, 95% CI: 0.99–51.94, *p* = 0.023; Figure 4G) subgroups and had no benefit on BCSS in the HR+/LN+ subgroup (hazard ratio = 1.15, 95% CI: 0.31–4.27, *p* = 0.840; Figure 4D). While after balancing the features between subgroups, chemotherapy among TC patients was also related to a worse BCSS in the HR+/LN− subgroup (PSM, hazard ratio = 6.95, 95% CI: 2.13–22.68, *p* = 0.001, Figure 4B; IPTW, hazard ratio = 3.04, 95% CI: 1.46–6.34, *p* = 0.003, Figure 4C) and showed no impact on BCSS in HR+/LN+ (PSM, hazard ratio = 0.75, 95% CI: 0.10–5.46, *p* = 0.780, Figure 4E; IPTW, hazard ratio = 0.80, 95% CI: 0.20–3.17, *p* = 0.755, Figure 4F) and HR−/LN− (PSM, hazard ratio = 7.87, 95% CI: 0.49–126.00, *p* = 0.150, Figure 4H; IPTW, hazard ratio = 2.18, 95% CI: 0.28–16.87, *p* = 0.457, Figure 4I).

**FIGURE 5:**
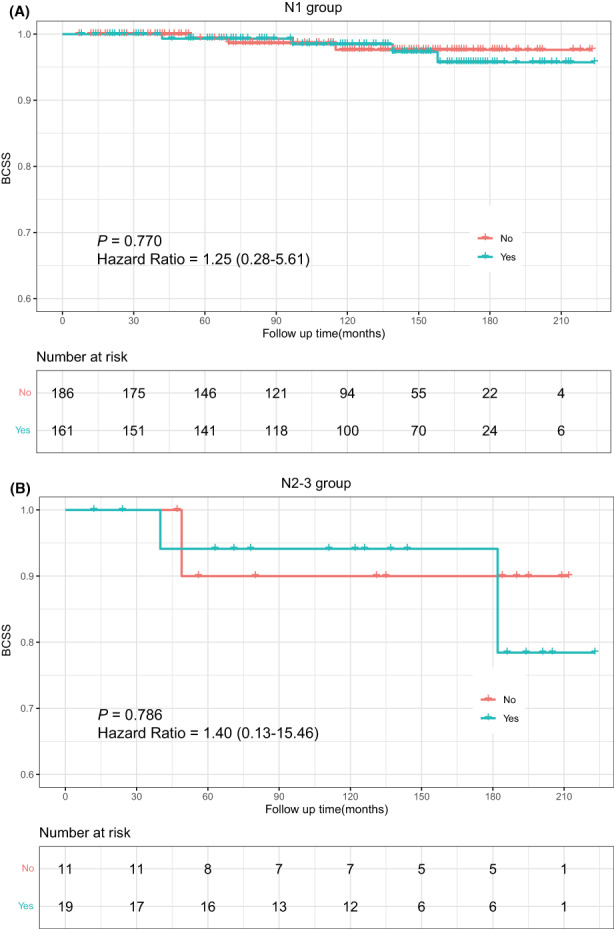
Prognostic effect of chemotherapy on tubular carcinoma patients with HR+/LN+ status in the N1 group (A) and N2–3 group (B). BCSS, breast cancer‐specific survival; HR, hormone receptor; LN, lymph node.

To further clarify whether N stage would affect the strategy of chemotherapy for the patients with positive HR status, the HR+/LN+ patients were stratified into N1 and N2–3 groups. Figure 5 illustrated that there was no difference between patients with chemotherapy and those with no/unknown chemotherapy in N1 stage (hazard ratio = 1.25, 95% CI: 0.28–5.61, *p* = 0.770, Figure 5A) and N2–3 stage (hazard ratio = 1.40, 95% CI: 0.13–15.46, *p* = 0.786, Figure 5B). Notably, since TC patients rarely experienced extensive LN involvement, there were too few subjects in the N2–3 group (*n* = 16) to be statistically significant.

**FIGURE 6:**
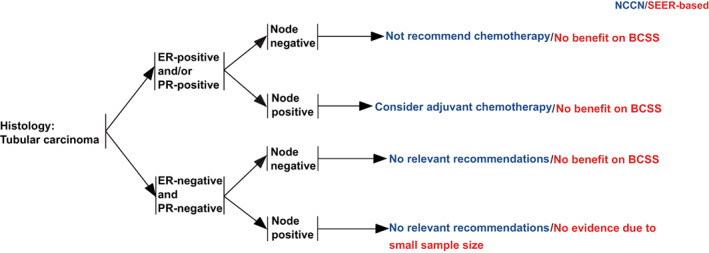
Recommendations on chemotherapy stratified by hormone receptor and lymph node status and comparison with NCCN guidelines for tubular carcinoma. BCSS, breast cancer‐specific survival; ER, estrogen receptor; NCCN, National Comprehensive Cancer Network; PR, progestogen receptor; SEER, Surveillance, Epidemiology, and End Results database.

Based on the results above, recommendations on chemotherapy could be provided for TC patients regarding the long‐term BCSS after being stratified by HR and LN status (Figure 6). For the HR+/LN− subgroup in our research, the 10‐year BCSS was as high as 100% and 98.9% in patients with no/unknown chemotherapy, while 97.4% and 97.3% in those with chemotherapy after PSM and IPTW, respectively, indicating that chemotherapy was not recommended for HR+/LN− patients, which was consistent with the current NCCN guidelines. The NCCN guidelines recommended that HR+/LN+ patients should consider adjuvant chemotherapy, while no benefit of chemotherapy was observed for HR+/LN+ patients based on our analysis. The benefit of chemotherapy was still not observed, although we stratified HR+/LN+ patients according to N stage. However, evidence on HR+/LN+ patients with N2–3 stage was limited due to a small sample size (*n* = 16), which called for future studies with more participants. Given that most TC patients had positive HRs, the current NCCN guideline did not give any recommendations for TC patients with negative HRs. According to this research, chemotherapy showed no benefit to HR−/LN− patients. Therefore, chemotherapy was not recommended either. However, analyses on HR−/LN+ patients were not performed because small samples (*n* = 4) could not provide sufficient statistical efficiency, which called for studies with a larger population.

## 4 DISCUSSION

Tubular carcinoma of the breast has long been considered to show predictors of favorable prognoses, such as small tumor size, low risk of LN metastasis, well differentiation, and high expression of HRs,^15^, ^16^, ^17^ and has been proven to have a lower risk of recurrence and better long‐term survival than IDC.^15^, ^18^, ^19^ Appropriate treatment regimens for TC should be made according to its clinicopathological characteristics and response to treatment. However, due to the low rate of 1%–4% in breast cancer,^14^, ^20^ extensive population studies are lacking to provide sufficient evidence for TC therapy. This study reviewed a large TC population derived from the SEER database, described their clinicopathological features and treatment information, explored the predictors of patients receiving chemotherapy, and stratified the study population by HR and LN status to evaluate the efficacy of chemotherapy on TC patients.

This study further confirmed that TC tended to show more favorable features than IDC, such as lower histological grade, smaller tumor size, less involved LNs, and a high positive rate of HRs, which was similar to earlier research.^16^, ^17^, ^19^ Similar to previous reports,^15^, ^18^ after using 1:1 PSM to balance the differences between groups, TC (10‐year BCSS, 98.4%) still showed better long‐term survival than IDC (10‐year BCSS, 97.3%). Sun et al.^11^ followed up 5677 postoperative patients with TC for 13.9 years, and only 74 cases of breast cancer‐related deaths were reported, and the 10‐year BCSS of the population was as high as 98%. The low invasive histological features and biological behavior of TC might predict its lower tendency of metastasis and excellent prognosis.

Molecular typing of breast cancer has been studied in recent years and plays a determining role in developing treatment strategies. TC, as has been reported in previous studies,^12^, ^14^, ^21^ belongs to the Luminal A subtype, of which immunohistochemistry and/or in situ hybridization usually show high expression of ER and PR, negative HER2 status, and a Ki‐67 proliferation rate less than 10%. Luminal A is a subtype with a favorable prognosis that shows low proliferation and poor response to chemotherapy, and it remains controversial whether breast cancer of Luminal A should receive chemotherapy.^22^, ^23^ In former practice, chemotherapy was not often recommended for TC,^10^, ^14^, ^24^ which showed typical features of the Luminal A subtype and a favorable prognosis. As reported in previous studies, only 9%–16.1% of TC patients received chemotherapy,^13^, ^19^, ^24^, ^25^ and in our study, only 7.4% (*n* = 494) of patients with TC underwent chemotherapy. TC patients receiving chemotherapy in the current study tended to be younger at diagnosis, have a higher histological grade, have larger tumors, have more involved LNs, and have a lower possibility of positive HR status than those without chemotherapy. However, evidence of chemotherapy on the Luminal A subtype is mainly based on IDC, and limited trials are available for TC.

Combined with the practice of the Luminal A subtype of breast cancer, TC, characterized as positive HR status, negative HER2 status, and low proliferation, tended not to benefit from chemotherapy.^22^, ^26^ Kitchen et al.^27^ conducted a long‐term follow‐up of 86 TC patients, which showed that the risk of death of these patients receiving chemotherapy was reduced by 85% compared with those without chemotherapy. However, due to the small sample size, the subgroup of TC patients with potential benefits of chemotherapy could not be further determined. To explore the efficacy of chemotherapy on TC, we performed survival analyses and adjusted for confounding factors among 6486 TC patients, which revealed that chemotherapy was an unfavorable predictor of BCSS for TC. The underlying reasons deserve further exploration.

According to our results, the majority of TC patients were of positive HR (98.7%) and negative LN (94.1%). Compared to patients receiving no/unknown chemotherapy, a worse long‐term prognosis among those who received chemotherapy was observed in the HR+/LN− group. TC of HR+/LN− is a subtype characterized by the Luminal A tumor on which the risk of side effects from chemotherapy might be greater than the benefit. Therefore, overtreatment of HR+/LN− patients could potentially yield adverse prognostic effects. A study of 264 subjects on neoadjuvant chemotherapy showed a lower pathological complete response rate for the Luminal A subtype (3.03%) than the average (12.50%).^28^ Nevertheless, TC patients who underwent only standard local treatment, including surgery and radiotherapy, achieved long‐term remission and the expected survival.^11^ Consequently, the therapeutic strategy for TC patients should be carefully developed. On the other hand, some worse clinicopathological features were found in HR+/LN− patients who received chemotherapy, such as more advanced grade, larger tumors, and less radiotherapy. Even if we balanced the differences between groups, there are other inevitable confounding biases that we failed to control. In the SEER database, patients with chemotherapy might have more co‐morbidities, did not receive R0 resection, and had tumors with non‐pure TC components, thus falsely concluding that their prognosis was worse.

According to the latest NCCN guidelines, TC patients with positive LNs should consider chemotherapy, so we further stratified patients to recognize potential candidates for chemotherapy. In our multivariable analysis, HR status and LN involvement were independent factors affecting the BCSS of TC. To provide individualized treatment recommendations and protect patients from unnecessary side effects of cytotoxic agents, we stratified TC patients by HR and LN status to determine whether there exist subgroups of TC sensitive to chemotherapy. However, no patients could benefit from chemotherapy in our subgroup analysis. After PSM and IPTW, patients with positive HR status derived no benefit from chemotherapy, whether with negative LN or positive LN status, and neither did HR−/LN− patients. Therefore, we believe that most TC patients did be exempt from chemotherapy, regardless of the HR and LN status. Although LN status has long been considered an essential factor of therapy, randomized clinical trials concluded that patients with node‐positive, HR‐positive breast cancer did not benefit from chemotherapy.^29^, ^30^ In a large population‐based study, LN involvement was an unfavorable predictor of the prognosis of TC, while TC patients with positive LNs were still not recommended to receive chemotherapy.^31^ However, tumors are heterogeneous, and there are individual differences between patients. Although clinical studies with large‐scale data provided important evidence for treatment, variables such as gene expression, sensitivity to endocrine therapy, risk factors, and economic status should be taken into account in the diagnosis and treatment process, and individualized treatment regimens should be adapted to decide whether to administer chemotherapy to patients with TC. Previous reports on TC yield similar findings that adjuvant chemotherapy was not beneficial to TC.^14^, ^19^, ^24^

ILC is another subtype of invasive breast cancer,^32^ which also has a high (90%) rate of HR positivity and shows a negative HER2 status.^5^, ^6^ Because of the sensitivity to endocrine therapy, some studies concluded that administering chemotherapy to ILC patients receiving endocrine therapy did not increase the survival benefit.^33^, ^34^ Therefore, similar to TC, it was controversial whether chemotherapy should be administered to ILC.^35^ However, there was no study comparing the prognosis of TC with those of ILC, and this study proved that TC (10‐year BCSS, 98.0%) had a better long‐term prognosis than ILC (10‐year BCSS, 95.3%) after IPTW. In this study, more than 98% of patients in grade I–II ILC and TC groups had positive HRs. However, the rate of LN involvement was significantly higher in ILC (32.0%) than in TC (5.9%) and also higher than in the IDC group (25.3%). Current treatment guidelines for ILC are the same as for IDC, with chemotherapy assessed by LN status. However, less LN involvement and a better prognosis of TC implied a different treatment modality.

Currently, the standard clinical treatment for TC of the breast is the comprehensive application of surgery, radiotherapy, endocrine therapy, and so on. In this study, TC patients proved to benefit from local treatment. Breast‐conserving surgery and mastectomy showed similar therapeutic efficacy, and radiotherapy was also favorable for TC. A large population‐based study by Özkurt E et al. suggested that the de‐escalation of surgical axillary staging should be considered for T1 TC.^4^ In our cohort, 95.9% of TC patients were staged T0–1, so de‐escalation of axillary surgery could be considered. The efficacy of endocrine therapy potentially affected the administration of chemotherapy as well as the prognosis of patients, which is a critical aspect in TC studies. However, we were unable to analyze the effect of endocrine therapy due to the limitation of data acquisition. According to several works in the literature, there also existed controversy about whether endocrine therapy should be performed on TC.^14^, ^19^, ^20^ Therefore, evidence on systematic treatment, including chemotherapy and endocrine therapy, still calls for further exploration in future clinical trials.

There were limitations in the current research. First, study with a retrospective design might bear potential biases, such as selection bias and loss to follow‐up bias. Second, the diagnosis of TC of the breast using the SEER database omitted central pathological review. On the one hand, the percentage of TC components could not be assured. On the other hand, the antibodies used and immunohistochemical staining might be interpreted differently in different centers involved, resulting in diagnoses potentially varying from person to person. Third, potentially important factors such as co‐morbidities, endocrine therapy information, and chemotherapy regimens were unavailable in this research. The effect of endocrine therapy was an essential factor in assessing the prognosis and administration of chemotherapy, and the chemotherapy protocol was also important information for evaluating the efficacy of chemotherapy. Therefore, the unavailability of data limited further research and the discussion of important aspects of this study. Fourth, other prognostic outcomes, such as progression‐free survival, could not be obtained from the SEER database, so they failed to be analyzed. Moreover, TC patients with negative HR and positive LN status failed to be analyzed due to the small sample size, and further research based on a large population is needed in the future.

## 5 CONCLUSIONS

In conclusion, TC of the breast is a low‐grade malignant tumor with excellent long‐term survival and is characterized by a well‐differentiated, early‐stage, and positive HR status. Adjuvant chemotherapy was not recommended for TC, regardless of HR and LN status. Still, regimens should be tailored for individual patients, and further studies are needed to guide the treatment of TC.

## AUTHOR CONTRIBUTIONS

**Yuting Zhao:** Conceptualization (lead); data curation (lead); writing – original draft (lead). **Na Chai:** Formal analysis (lead); methodology (equal); writing – original draft (equal). **Shouyu Li:** Software (lead); visualization (lead). **Lutong Yan:** Investigation (lead); validation (lead). **Can Zhou:** Methodology (equal); resources (lead). **Jianjun He:** Project administration (lead); supervision (equal). **Huimin Zhang:** Conceptualization (equal); supervision (lead); writing – review and editing (lead).

## CONFLICT OF INTEREST STATEMENT

The authors declare that they have no competing interests.

## ETHICS STATEMENT

This study was approved by the Ethical Committee of the First Affiliated Hospital of Xi'an Jiaotong University. The SEER data erase the identity information of patients, so there is no need for informed consent from the patients.

## Supporting information


Appendix S1.

## Data Availability

The datasets analyzed during the current study are available in the SEER Program (www.seer.cancer.gov) SEER*Stat Database: Incidence‐SEER 18 Regs Research Data + Hurricane Katrina Impacted Louisiana Cases, Nov 2017 Sub (1973–2015 varying).
