# Supplementary material for: Evaluation of the efficacy of chemotherapy for tubular carcinoma of the breast: A Surveillance, Epidemiology, and End Results cohort study
Source: Cancer Med. 2023 Mar 6;12(9):10326–39. doi: 10.1002/cam4.5763 (PMC10225174; doi:10.1002/cam4.5763)
Supplement: Supplementary file 1 — Appendix S1. [file CAM4-12-10326-s001.pdf]

Supplementary Figure S1 Comparison of BCSS between tubular carcinoma treated with or without chemotherapy. BCSS, breast cancer-specific survival; TC, tubular carcinoma.

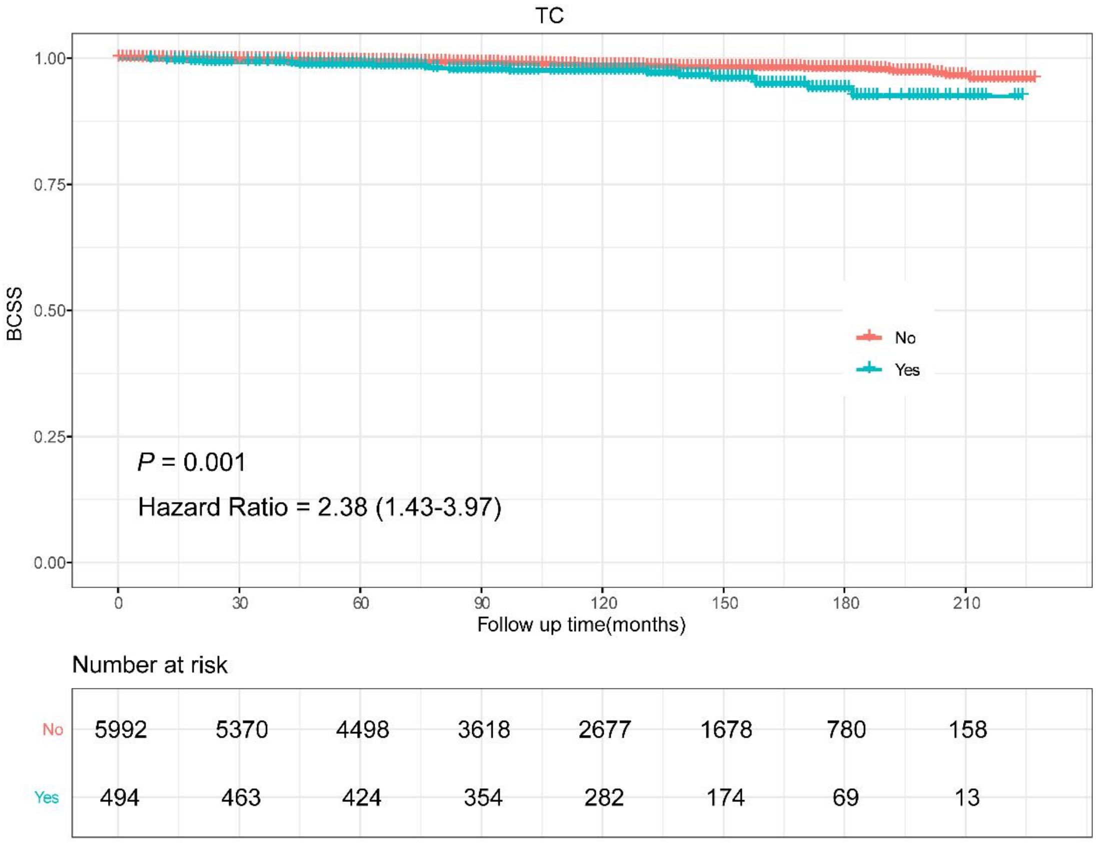

Supplementary Table S1 Clinicopathological characteristics of breast cancer patients diagnosed with ILC or TC in the original cohort, PSM cohort, and IPTW cohort.

Covariates	Original Cohort				PSM Cohort				IPTW Cohort			
	TC (%) n = 6,486	ILC (%) n = 46,972	*P* value	SMD	TC (%) n = 4,225	ILC (%) n = 4,225	*P* value	SMD	TC (%) n = 4,480	ILC (%) n = 4,862	*P* value	SMD
**Age at diagnosis**			<0.001	0.231			<0.001	0.300			0.002	0.063
<40	105 (1.6)	597 (1.3)			56 (1.3)	97 (2.3)			65 (1.4)	72 (1.4)		
40-64	3,958 (61.0)	23,486 (50.0)			2,006 (47.5)	2,581 (61.1)			2,495 (55.7)	2,858 (58.8)		
≥65	2,423 (37.4)	22,889 (48.7)			2,163 (51.2)	1,547 (36.6)			1,920 (42.9)	1,932 (39.7)		
**Race**			<0.001	0.207			<0.001	0.131			0.644	0.016
White	5,476 (84.4)	40,912 (87.1)			3,656 (86.5)	3,462 (81.9)			3,833 (85.6)	4,141 (85.2)		
Black	310 (4.8)	3,390 (7.2)			222 (5.3)	333 (7.9)			247 (5.5)	264 (5.4)		
Others	700 (10.8)	2,670 (5.7)			347 (8.2)	430 (10.2)			400 (8.9)	457 (9.4)		
**Marital status**			<0.001	0.069			0.123	0.045			0.801	0.012
Married	3,882 (59.9)	26,509 (56.4)			2,400 (56.8)	2,477 (58.6)			2,616 (58.4)	2,867 (59.0)		
Unmarried/ Loss of marriage	2,354 (36.3)	18,493 (39.4)			1,637 (38.7)	1,547 (36.6)			1,667 (37.2)	1,786 (36.7)		
Unknown	250 (3.9)	1,970 (4.2)			188 (4.4)	201 (4.8)			196 (4.4)	209 (4.3)		
**Grade**			<0.001	1.540			0.122	0.034			0.009	0.040
I	5,982 (92.2)	15,615 (33.2)			3,721 (88.1)	3,673 (86.9)			3,976 (88.7)	4,375 (90.0)		
II	504 (7.8)	31,357 (66.8)			504 (11.9)	552 (13.1)			504 (11.3)	487 (10.0)		
**T Stage**			<0.001	1.107			0.010	0.066			0.376	0.021

0-1	6,223 (95.9)	25,388 (54.0)			3,962 (93.8)	3,894 (92.2)			4,217 (94.1)	4,600 (94.6)		
2	221 (3.4)	15,352 (32.7)			221 (5.2)	268 (6.3)			221 (4.9)	220 (4.5)		
3-4	42 (0.6)	6,232 (13.3)			42 (1.0)	63 (1.5)			42 (0.9)	42 (0.9)		
**N Stage**			<0.001	0.724			<0.001	0.142			0.777	0.017
0	6,105 (94.1)	31,947 (68.0)			3,854 (91.2)	3,699 (87.6)			4,113 (91.8)	4,485 (92.3)		
1	351 (5.4)	9,620 (20.5)			341 (8.1)	441 (10.4)			337 (7.5)	346 (7.1)		
2	23 (0.4)	3,032 (6.5)			23 (0.5)	63 (1.5)			23 (0.5)	24 (0.5)		
3	7 (0.1)	2,373 (5.1)			7 (0.2)	22 (0.5)			7 (0.2)	7 (0.1)		
**HR Status**			<0.001	0.053			0.432	0.019			0.829	0.004
Positive	6,404 (98.7)	46,066 (98.1)			4,177 (98.1)	4,168 (98.7)			4,425 (98.8)	4,805 (98.8)		
Negative	82 (1.3)	906 (1.9)			48 (1.1)	57 (1.3)			55 (1.2)	57 (1.2)		
**Surgery**			<0.001	0.528			<0.001	0.121			0.080	0.030
Mastectomy	5,024 (77.5)	24,967 (53.1)			2,763 (65.4)	3,001 (71.0)			3,018 (67.4)	3,307 (66.0)		
BCT	1,462 (22.5)	22,005 (46.8)			1,462 (34.6)	1,224 (29.0)			1,462 (32.6)	1,655 (34.0)		
**Radiotherapy**			<0.001	0.167			<0.001	0.344			<0.001	0.114
No/unknown	2,676 (41.3)	23,278 (49.6)			2,626 (62.2)	3,282 (77.7)			2,573 (57.4)	3,062 (63.0)		
Yes	3,810 (58.7)	23,697 (50.4)			1,599 (37.8)	943 (22.3)			1,907 (42.6)	1,800 (37.0)		
**Chemotherapy**			<0.001	0.641			0.001	0.070			<0.001	0.114
No/unknown	5,992 (92.4)	31,983 (68.1)			3,754 (88.9)	3,657 (86.6)			4,018 (89.7)	4,379 (90.1)		
Yes	494 (7.6)	14,989 (31.9)			471 (11.1)	568 (13.4)			462 (10.3)	483 (9.9)		

Abbreviations: BCT, breast-conserving therapy; HR, hormone receptor; ILC, invasive lobular carcinoma; IPTW, inverse probability of treatment weighting; PSM, propensity score matching; SMD, standardized mean difference; TC, tubular carcinoma.

Supplementary Table S2 Multivariate analysis of breast cancer-specific survival predictors in the original cohort using the Cox proportional hazard model.

Covariates	Original Cohort		
	Hazard ratio	*P* value	95% CI
**Age at diagnosis**			
<40	Reference		
40-64	0.71	<0.001	0.67-0.75
≥65	1.17	<0.001	1.10-1.25
**Race**			
White	Reference		
Black	1.37	<0.001	1.32-1.43
Others	0.88	<0.001	0.84-0.92
**Marital status**			
Married	Reference		
Unmarried/ Loss of marriage	1.29	<0.001	1.25-1.32
Unknown	1.19	<0.001	1.10-1.28
**Subtype**			
TC	Reference		
IDC	1.84	<0.001	1.51-2.25
ILC	2.01	<0.001	1.64-2.46
**Grade**			
I	Reference		
II	1.70	<0.001	1.64-1.77
**T Stage**			
0-1	Reference		
2	2.09	<0.001	2.03-2.16
3-4	3.48	<0.001	3.32-3.64
**N Stage**			
0	Reference		
1	1.86	<0.001	1.79-1.92
2	3.37	<0.001	3.21-3.53
3	6.27	<0.001	5.96-6.61
**HR Status**			
Positive	Reference		
Negative	2.20	<0.001	2.11-2.29
**Surgery**			
Mastectomy	Reference		
BCT	1.08	<0.001	1.05-1.11
**Radiotherapy**			
No/unknown	Reference		

Yes	0.69	<0.001	0.67-0.72
**Chemotherapy**			
No/unknown	Reference		
Yes	1.02	0.253	0.99-1.06

Abbreviations: BCT, breast conserving therapy; CI, confidence interval; HR, hormone receptor; IDC, invasive ductal carcinoma; ILC, invasive lobular carcinoma; TC, tubular carcinoma.

Supplementary Table S3 Clinicopathological characteristics of tubular carcinoma patients treated with and without chemotherapy.

Covariates	TC		
	No/Unk Chemotherapy (%) n = 5,992	Chemotherapy (%) n = 494	*P* value
**Age at diagnosis**			< 0.001
<40	70 (1.2)	35 (7.1)	
40-64	3,574 (59.6)	384 (77.7)	
≥65	2,348 (39.2)	75 (15.2)	
**Race**			< 0.001
White	5,093 (85.0)	383 (77.5)	
Black	270 (4.5)	40 (8.1)	
Others	629 (10.5)	71 (14.4)	
**Marital status**			0.011
Married	3,558 (59.4)	324 (65.6)	
Unmarried/Loss of marriage	2,195 (36.6)	159 (32.2)	
Unknown	239 (4.0)	11 (2.2)	
**Grade**			< 0.001
I	5,561 (92.8)	421 (85.2)	
II	431 (7.2)	73 (14.8)	
**T Stage**			< 0.001
0-1	5,819 (97.1)	404 (81.8)	
2	139 (2.3)	82 (16.6)	
3-4	34 (0.6)	8 (1.6)	
**N Stage**			< 0.001
0	5,791 (96.6)	314 (63.6)	
1	190 (3.2)	161 (32.6)	
2	8 (0.1)	15 (3.0)	
3	3 (0.1)	4 (0.8)	
**HR Status**			0.002
Positive	5,924 (98.9)	480 (97.2)	
Negative	68 (1.1)	14 (2.8)	
**Surgery**			< 0.001
Mastectomy	4,701 (78.5)	323 (65.4)	
BCT	1,291 (21.5)	171 (34.6)	
**Radiotherapy**			0.228
No/unknown	2,459 (41.0)	217 (43.9)	
Yes	3,533 (59.0)	277 (56.1)	

Abbreviations: BCT, breast conserving therapy; HR, hormone receptor; TC, tubular carcinoma; Unk, unknown.

Supplementary Table S4 Clinicopathological characteristics of tubular carcinoma patients with different status of hormone receptor and lymph node.

Covariates	HR+LN-			HR+LN+			HR-LN-		
	No/Unk Chemo (%) n = 5,725	Chemo (%) n = 302	*P* value	No/Unk Chemo (%) n = 199	Chemo (%) n = 178	*P* value	No/Unk Chemo (%) n = 66	Chemo (%) n = 12	*P* value
**Age at diagnosis**			<0.001			<0.001			0.029
<40	67 (1.2)	23 (7.6)		3 (1.5)	11 (6.2)		0 (0.0)	1 (8.3)	
40-64	3,424 (59.8)	236 (78.1)		109 (54.8)	137 (77.0)		41 (62.1)	9 (75.0)	
≥65	2,234 (39.0)	43 (14.2)		87 (43.7)	30 (16.9)		25 (37.9)	2 (16.7)	
**Race**			0.001			0.191			0.320
White	4,875 (85.2)	233 (77.2)		161 (80.9)	141 (79.2)		56 (84.8)	8 (66.7)	
Black	255 (4.5)	21 (7.0)		10 (5.0)	17 (9.6)		5 (7.6)	2 (16.7)	
Others	595 (10.4)	48 (15.9)		28 (14.1)	20 (11.2)		5 (7.6)	2 (16.7)	
**Marital status**			0.260			0.010			0.807
Married	3,407 (59.5)	194 (64.2)		114 (57.3)	121 (68.0)		35 (53.0)	7 (58.3)	
Unmarried/ Loss of marriage	2,091 (36.5)	98 (32.5)		75 (37.7)	56 (31.5)		29 (43.9)	5 (41.7)	
Unknown	227 (4.0)	10 (3.3)		10 (5.0)	1 (0.6)		2 (3.0)	0 (0.0)	
**Grade**			<0.001			1.000			0.642
I	5,327 (93.0)	255 (84.4)		172 (86.4)	154 (86.5)		61 (92.4)	10 (83.3)	
II	398 (7.0)	47 (15.6)		27 (13.6)	24 (13.5)		5 (7.6)	2 (16.7)	
**T Stage**			<0.001			0.021			0.040

0-1	5,582 (97.5)	258 (85.4)		172 (86.4)	135 (75.8)		64 (97.0)	10 (83.3)	
2	111 (1.9)	41 (13.6)		26 (13.1)	39 (21.9)		2 (3.0)	1 (8.3)	
3-4	32 (0.6)	3 (1.0)		1 (0.5)	4 (2.2)		0 (0.0)	1 (8.3)	
**Surgery**			<0.001			0.074			0.081
Mastectomy	4,531 (79.1)	196 (64.9)		113 (56.8)	118 (66.3)		56 (84.8)	7 (58.3)	
BCT	1194 (20.9)	106 (35.1)		86 (43.2)	60 (33.7)		10 (15.2)	5 (41.7)	
**Radiotherapy**			0.030			0.001			0.900
No/unknown	2,321 (40.5)	142 (47.0)		113 (56.8)	70 (39.3)		23 (34.8)	5 (41.7)	
Yes	3,404 (59.5)	160 (53.0)		86 (43.2)	108 (60.7)		43 (65.2)	7 (58.3)	

Abbreviations: BCT, breast conserving therapy; Chemo, chemotherapy; HR, hormone receptor; LN, lymph node; Unk, unknown.

Supplementary Table S5 Clinicopathological characteristics of tubular carcinoma patients with different status of hormone receptor and lymph node after 1:1 PSM.

Covariates	HR+LN-			HR+LN+			HR-LN-		
	No/Unk Chemo (%) n = 299	Chemo (%) n = 299	*P* value	No/Unk Chemo (%) n = 125	Chemo (%) n = 125	*P* value	No/Unk Chemo (%) n = 9	Chemo (%) n = 9	*P* value
**Age at diagnosis**			0.896			0.435			1.000
<40	24 (8.0)	21 (7.0)		3 (2.4)	1 (0.8)		0 (0.0)	0 (0.0)	
40-64	232 (77.6)	235 (78.6)		88 (70.4)	95 (76.0)		8 (88.9)	7 (77.8)	
≥65	43 (14.4)	43 (14.4)		34 (27.2)	29 (23.2)		1 (11.1)	2 (22.2)	
**Race**			0.929			0.180			0.445
White	232 (77.6)	232 (77.6)		102 (81.6)	99 (79.2)		8 (88.9)	6 (66.7)	
Black	18 (6.0)	20 (6.7)		5 (4.0)	12 (9.6)		1 (11.1)	2 (22.2)	
Others	49 (16.4)	47 (15.7)		18 (14.4)	14 (11.2)		0 (0.0)	1 (11.1)	
**Marital status**			0.830			0.726			0.635
Married	191 (63.9)	194 (64.9)		84 (67.2)	78 (62.4)		6 (66.7)	4 (44.4)	
Unmarried/ Loss of marriage	100 (33.4)	95 (31.8)		40 (32.0)	46 (36.8)		3 (33.3)	5 (55.6)	
Unknown	8 (2.7)	10 (3.3)		1 (0.8)	1 (0.8)				
**Grade**			0.910			1.000	0 (0.0)	0 (0.0)	0.453
I	252 (84.3)	254 (84.9)		112 (89.6)	112 (89.6)		9 (100.0)	7 (77.8)	
II	47 (15.7)	45 (15.1)		13 (10.4)	13 (10.4)		0 (0.0)	2 (22.2)	
**T Stage**			0.116			0.562			1.000

0-1	266 (89.0)	258 (86.3)		105 (84.0)	108 (86.4)		8 (88.9)	9 (100.0)	
2	27 (9.0)	39 (13.0)		19 (15.2)	17 (13.6)		1 (11.1)	0 (0.0)	
3-4	6 (2.0)	2 (0.7)		1 (0.8)	0 (0.0)		0 (0.0)	0 (0.0)	
**Surgery**			0.931			0.894			1.000
Mastectomy	198 (66.2)	196 (65.6)		81 (64.8)	83 (66.4)		6 (66.7)	6 (66.7)	
BCT	101 (33.8)	103 (34.4)		44 (35.2)	42 (33.6)		3 (33.3)	3 (33.3)	
**Radiotherapy**			1.000			0.611			1.000
No/unknown	138 (46.2)	139 (46.5)		58 (46.4)	53 (42.4)		3 (33.3)	3 (33.3)	
Yes	161 (53.8)	160 (53.5)		67 (53.6)	72 (57.6)		6 (66.7)	6 (66.7)	

Abbreviations: BCT, breast conserving therapy; Chemo, chemotherapy; HR, hormone receptor; LN, lymph node; PSM, propensity score matching; Unk, unknown.

Supplementary Table S6 Clinicopathological characteristics of tubular carcinoma patients with different status of hormone receptor and lymph node after IPTW.

Covariates	HR+LN-			HR+LN+			HR-LN-		
	No/Unk Chemo (%) n = 303	Chemo (%) n = 304	*P* value	No/Unk Chemo (%) n = 124	Chemo (%) n = 121	*P* value	No/Unk Chemo (%) n = 10	Chemo (%) n = 10	*P* value
**Age at diagnosis**			0.980			0.972			0.870
<40	21 (6.9)	21 (6.7)		3 (2.4)	3 (2.2)		0 (0.0)	0 (0.0)	
40-64	236 (77.9)	238 (78.5)		90 (72.6)	89 (73.4)		8 (80.0)	8 (80.0)	
≥65	46 (15.2)	45 (14.8)		31 (25.0)	29 (24.4)		2 (20.0)	2 (20.0)	
**Race**			0.973			0.962			0.980
White	236 (77.9)	236 (77.6)		99 (79.4)	96 (79.1)		7 (70.0)	7 (70.0)	
Black	21 (6.9)	21 (6.9)		8 (7.0)	8 (6.4)		2 (20.0)	2 (20.0)	
Others	46 (15.2)	47 (15.5)		17 (13.6)	17 (14.5)		1 (10.0)	1 (10.0)	
**Marital status**			0.995			0.994			0.938
Married	195 (64.5)	197 (64.8)		82 (65.9)	80 (66.1)		5 (50.0)	5 (50.0)	
Unmarried/ Loss of marriage	98 (32.2)	97 (31.9)		41 (33.3)	40 (33.1)		5 (50.0)	5 (50.0)	
Unknown	10 (3.3)	10 (3.3)		1 (0.8)	1 (0.8)		0 (0.0)	0 (0.0)	
**Grade**			0.861			0.895			0.792
I	257 (84.8)	259 (85.2)		109 (87.9)	106 (87.6)		8 (80.0)	8 (80.0)	
II	46 (15.2)	45 (14.8)		15 (12.1)	15 (12.4)		2 (20.0)	2 (20.0)	
**T Stage**			0.966			0.933			0.763

0-1	264 (87.1)	263 (86.5)		104 (83.9)	102 (84.4)		9 (90..0)	9 (90.0)	
2	36 (12.0)	38 (12.5)		19 (15.3)	18 (15.0)		1 (10.0)	1 (10.0)	
3-4	3 (0.9)	3 (1.0)		1 (0.8)	1 (0.6)		0 (0.0)	0 (0.0)	
**Surgery**			0.920			0.995			0.941
Mastectomy	200 (66.0)	199 (65.5)		77 (62.1)	76 (62.8)		6 (60.0)	6 (60.0)	
BCT	103 (34.0)	105 (34.5)		47 (37.9)	45 (37.2)		4 (40.0)	4 (40.0)	
**Radiotherapy**			0.961			0.887			0.928
No/unknown	139 (45.9)	140 (46.1)		59 (47.6)	57 (47.1)		4 (40.0)	4 (40.0)	
Yes	164 (54.1)	164 (53.9)		65 (52.4)	64 (52.9)		6 (60.0)	6 (60.0)	

Abbreviations: BCT, breast conserving therapy; Chemo, chemotherapy; HR, hormone receptor; IPTW, inverse probability of treatment weighting; LN, lymph node; Unk, unknown.
